# Nutrition in Educational Institutions—The Perspective of School Principals and Parents on the Tasks of Local Governments (Poland)

**DOI:** 10.3390/nu16183214

**Published:** 2024-09-23

**Authors:** Karolina Sobczyk, Karolina Krupa-Kotara, Marlena Robakowska, Jarosław Markowski, Mateusz Grajek

**Affiliations:** 1 Department of Economics and Health Care Management, Faculty of Public Health in Bytom, Medical University of Silesia in Katowice, 40-007 Katowice, Poland; ksobczyk@sum.edu.pl; 2 Department of Epidemiology, Faculty of Public Health in Bytom, Medical University of Silesia in Katowice, 41-902 Bytom, Poland; kkrupa@sum.edu.pl; 3 Department of Public Health and Social Medicine, Medical University of Gdansk, 80-210 Gdansk, Poland; marlena.robakowska@gumed.edu.pl; 4 Department of Laryngology, Faculty of Medical Sciences in Katowice, Medical University of Silesia in Katowice, 40-007 Katowice, Poland; jmarkowski@sum.edu.pl; 5 Department of Public Health, Faculty of Public Health in Bytom, Medical University of Silesia in Katowice, 41-902 Bytom, Poland

**Keywords:** school nutrition, local governments, parent satisfaction, meal quality, nutrition education

## Abstract

Background: The financing of nutrition in educational institutions is one of the cornerstones of the activities of local governments in Poland. Proper management of this area is crucial to ensuring that children and young people have access to wholesome meals, which directly affects their health, physical development, as well as educational effectiveness. Objective: This study aimed to examine how school principals and parents perceive the role of local governments in managing school nutrition in Poland, given the growing importance of nutrition programs for children’s health. Methods: A survey was conducted with 200 school principals and 1000 parents, assessing satisfaction with the implementation, quality, and organization of nutrition programs overseen by local authorities. Results: The results revealed significant differences between the two groups. While 75% of principals expressed satisfaction with the quality of meals provided in schools, only 55% of parents shared this view. Similarly, 80% of principals rated the organization of cooperation with food suppliers positively, compared to only 50% of parents. Regarding allergen information, 65% of principals felt adequately informed, while only 30% of parents agreed. These differences were statistically significant in several key areas, including adherence to nutrition standards (*p* = 0.009), frequency of health inspections (*p* = 0.009), and availability of allergen information (*p* = 0.013). Conclusions: The findings highlight a need for improved communication and collaboration between schools, parents, and local governments to enhance the effectiveness of nutrition programs. It is recommended that regular informational meetings be held and that the flow of information be improved to increase parental satisfaction and the overall effectiveness of school nutrition initiatives.

## 1. Background

The financing of nutrition in educational institutions is one of the cornerstones of the activities of local governments in Poland. Proper management of this area is crucial to ensuring that children and young people have access to wholesome meals, which directly affects their health, physical development, as well as educational effectiveness. This process is extremely complex and requires close cooperation between local government units and central authorities, as well as institutions that control the quality and standards of nutrition [1].

The legal basis that regulates nutrition in educational institutions is the Regulation of the Minister of Health of 26 July 2016 [2]. This document specifies the requirements for groups of foodstuffs that can be offered to children as part of mass nutrition in schools and kindergartens. This regulation plays a key role in shaping nutrition policy at the local level, ensuring consistency in the actions taken by various local government units [3].

Another important document is the Decree of the Minister of Education and Science of 30 March 2023, which introduces detailed guidelines for the organization and financing of nutrition in educational institutions. Among other things, the regulations specify procedures for granting and accounting for targeted subsidies intended to subsidize nutrition, with the aim of optimizing the financing process and ensuring greater transparency in the management of public funds [4].

Funding for nutrition in educational institutions is mainly provided by the local government’s own funds, which are supplemented by targeted subsidies from the state budget. In 2023, a significant portion of these funds came from the Assistance Fund, which was extended by an amendment to the law on 14 December 2022. This fund plays a key role in ensuring the financial stability of nutrition-related activities in educational institutions, enabling local governments to more effectively plan their budgets for future years [5].

Local government units are required to determine the procedure for granting and accounting for subsidies, which is regulated at the local level through resolutions of municipal and county councils. In practice, this means that TSUs must develop internal regulations that specify how nutrition funds will be allocated and what criteria establishments must meet in order to benefit from them. This approach allows for flexible adaptation of nutrition policies to the specifics of local needs and financial capabilities [6].

Nutrition standards in educational institutions are defined in detail in current regulations. According to them, menus must be varied and meet nutritional standards for different age groups. Key recommendations include serving vegetables and fruits at every meal, limiting fried foods, and ensuring an adequate supply of whole-grain cereals and fish. This approach aims not only to meet the basic nutritional needs of children but also to form healthy eating habits from an early age [7].

Supervision of the quality of nutrition in educational institutions is exercised by the authorities of the State Sanitary Inspection Service (PIS), which regularly inspects whether the meals provided meet legal requirements and nutritional standards. If irregularities are found, establishments must immediately take corrective action, which often involves changing food suppliers, modifying menus, or training kitchen staff [8].

Despite clearly defined legal regulations, the implementation of tasks related to the financing and organization of nutrition in educational institutions faces numerous challenges. One of the main problems is the rising cost of food, which is due to global market trends and inflation. The increase in the price of food raw materials directly affects the budgets of local government units, which have to look for additional sources of funding or make savings in other areas of activity [2].

Another challenge is the need to adapt to changing nutritional standards and growing public expectations for meal quality. Modern approaches to nutrition in educational institutions are increasingly incorporating ecological aspects, such as reducing food waste and promoting local and organic products. However, the introduction of such changes requires adequate preparation at both the administrative and practical levels, which places an additional burden on local governments [7].

An important element that can improve the quality of nutrition in educational institutions is education on healthy eating. This includes not only the training of staff responsible for preparing meals but also activities aimed at students themselves and their parents. Creating nutritional awareness among children and adolescents is key to changing eating habits, which in the long run can contribute to improving public health [6].

In conclusion, the financing of nutrition in educational institutions by local governments is a multifaceted process, requiring not only adequate financial resources but also effective management and close cooperation between different levels of public administration. In the face of growing challenges, it is necessary to constantly seek new solutions to provide children and young people with meals of the highest quality, in accordance with current nutritional and health standards.

The research hypotheses are the following: (1) there is concordance between the ratings of school principals and students’ parents on local governments’ implementation of nutrition-related tasks, but parents report lower levels of satisfaction overall; (2) school principals rate the quality and organization of nutrition (including meal quality, adherence to nutrition standards, and frequency of sanitary inspections) higher than parents, who are more critical in these areas; (3) parents of students are more likely to report dissatisfaction with operational aspects, such as schools’ cooperation with suppliers, availability of allergy information, and involvement in nutrition education, compared to school management ratings.

Thus, the purpose of the survey was to analyze in detail and assess the satisfaction of school principals and parents of students on the implementation by local governments of tasks related to the nutrition of children and adolescents in educational institutions in Poland. The survey aims to identify the level of agreement or discrepancy in the assessments of the two groups of respondents with regard to various aspects related to the organization, quality, and availability of nutrition in schools. In addition, the survey aims to identify areas that need to be improved in order to better meet the expectations of both school management and parents, as well as to understand how differences in perception may affect the implementation of nutrition policies at the local level.

## 2. Material and Methods

### 2.1. Study Group

The survey was cross-sectional. Data were collected at a single point in time through questionnaires to school principals and parents. The survey included an assessment of the current situation (2024) regarding the quality of nutrition and meal arrangements in schools. The survey was conducted on a representative sample of 200 school principals and 1000 parents of students from different regions of Poland. The selection of the sample was guided by the desire to obtain the greatest possible diversity in terms of geographic location, size of locality, type of school (elementary, middle, and high school), and socioeconomic diversity of respondents. For the study, 200 schools were selected from a total of about 2500 public schools in Poland. The selection of schools was random and carried out using a computer algorithm. Among the parents of students at these schools, 1000 were randomly selected from a total of about 50,000 potential participants. The sample size was calculated taking into account data from the Central Statistical Office, assuming a confidence level of 95% and a maximum measurement error of 5%, which ensures high reliability of the results.

To ensure the reliability and representativeness of the survey results, a random sampling method was used. The survey included school principals and parents of students from all over the country. First, a list of all primary and secondary schools in the country was compiled, as well as lists of students in those schools. From this list, a representative sample of schools was randomly selected using a computer random sampling algorithm, ensuring that each school had an equal chance of being selected. Principals at each selected school were then contacted and invited to participate in the survey. For the sample of principals, a group of parents of students in each school was randomly selected and invited to complete the surveys. To ensure that the results were representative, the sample included schools from different regions and types. If necessary, additional random draws were made to complete the samples and increase their representativeness. All data collected were verified for accuracy, and the survey was conducted in accordance with ethical principles, ensuring the anonymity and confidentiality of respondents. This approach ensured reliable results that reflect the actual opinions of school principals and parents nationwide.

The representativeness of the sample was calculated using data from the Central Statistical Office on the number of schools and the number of students in Poland. Assuming a confidence level of 95% and a maximum measurement error of 5%, a sample of 1000 parents and 200 school principals was calculated to be sufficient to produce results representative of the entire population of student parents and school principals in Poland. The distribution of the sample mirrored the proportions found in the actual population, further increasing the reliability of the results.

### 2.2. Research Tool

The questionnaire used in the survey consisted of 15 closed-ended and semi-open-ended questions, covering aspects such as meal quality, meal organization and availability, cooperation with suppliers, nutrition education, and school nutrition management. The questions were divided into sections on the following:

Meal quality—included assessments of taste, nutritional value, variety of meals, and compliance with nutrition standards.

Nutrition organization and availability—addressed the availability of meals for all students, hours of serving, and ease of access to menu information.

Cooperation with food suppliers—assessed the quality and timeliness of deliveries, variety of products, and level of satisfaction with suppliers’ services.

Nutrition education—educational programs, the school’s commitment to promoting healthy lifestyles, and information about the dangers of poor nutrition were evaluated.

Food management by the school—questions addressed the effectiveness of budget management, availability of staff and infrastructure, and adherence to hygiene procedures.

The validation process included the following steps:

- Content Validity: Experts in education, dietetics, and sociology reviewed the questionnaire to ensure that the questions covered all relevant aspects of school nutrition. This ensured that the questionnaire was comprehensive and relevant to the study of the problem.
- Construct Validity: To make sure that the questionnaire actually measures the concepts it was intended to measure (e.g., quality of nutrition, satisfaction with food organization), a factor analysis was applied. As a result of this analysis, it was confirmed that the questions do indeed group into appropriate categories, confirming the construct’s validity.
- Criterion Validity: The questionnaire was compared with other previously validated survey tools used in research on nutrition in educational institutions. Correlations between scores from our questionnaire and scores from these tools were high, confirming criterion validity.
- Internal Consistency: Cronbach’s alpha coefficient was used to assess the consistency of responses to questions within each section of the questionnaire. The alpha values for each section of the questionnaire ranged from 0.78 to 0.91, indicating high internal consistency and reliability of the tool.

### 2.3. Ethical Standards

This study was conducted in accordance with all applicable ethical standards. Prior to the start of the study, consent was obtained from the ethics committee, and each participant was informed about the purpose of the study, its conduct, and the full voluntariness of participation. The anonymity of the respondents and the confidentiality of the data collected were also ensured, allowing for honest and reliable responses.

This study was conducted in accordance with the guidelines of the Declaration of Helsinki. Prior to the study, approval was obtained from the Bioethics Committee of the Medical University of Silesia in Katowice (consent no. PCN/CBN/0052/KB/127/22, approve date is 12.07.2022). Participants were informed about the purpose of the study and its voluntary nature and were assured of anonymity and confidentiality.

### 2.4. Statistical Tools

A number of statistical tools were used to analyze the collected data. Pearson’s correlation was used to assess the relationship between the evaluations of school management and parents of students in the various categories of the survey, identifying the extent to which the opinions of the two groups converge or diverge. Statistical significance tests (*p*-level) were conducted to determine whether the observed differences in ratings are statistically significant, with a significance level of α = 0.05 assumed, meaning that the significance of the results can be established with 95% confidence if the *p*-level is less than 0.05. Factor analysis was used to verify the validation of the questionnaire’s construct, allowing for the identification of the main factors influencing respondents’ perceptions of nutrition quality. In addition, arithmetic means and standard deviations were calculated to describe the central tendency and variability of ratings in each category, allowing a thorough understanding of the distribution of opinions in the study group.

## 3. Results

### 3.1. Satisfaction Level of School Management

The survey results on local government performance satisfaction reveal that 65% of school principals are satisfied or very satisfied, though 15% express dissatisfaction, indicating areas for improvement. Meal quality is rated positively, with 80% of principals evaluating it as good or very good, and only 5% expressing dissatisfaction. School nutrition funding is considered sufficient by 60% of directors, with 10% reporting insufficiency. Cooperation with local governments is rated positively by 80% of principals, while 5% view it negatively. Nutritional norm adherence is high, with 95% of schools reporting frequent compliance. Regular sanitary inspections occur in 80% of schools, though 5% report infrequent checks. Local government involvement in nutrition education is mostly rated as positive (60%), with no reports of extremely low involvement. Cooperation with food suppliers is rated as good or very good by 85% of executives, with only 5% dissatisfied. Information on food allergies is positively rated by 85%, and healthy eating programs are widely implemented, with 90% of schools adhering to them frequently. Local government responses to problems are generally swift, with 70% rating them as fast or very fast, though 28% indicate slower responses. Satisfaction with feeding programs for children from poor families is high (80%) but 5% express dissatisfaction. Cafeteria staff sufficiency is rated adequate by 55%, though 15% report insufficiency, highlighting potential staffing needs. Kitchen infrastructure satisfaction is high, with 70% of directors satisfied but 10% dissatisfied. Dietary adaptations, such as vegetarian or gluten-free meals, are frequently offered in 75% of schools, but 25% report infrequent or absent adaptations, suggesting a need for further dietary accommodation (Table 1).

### 3.2. Parents’ Satisfaction Level

The survey results on parents’ satisfaction with local government performance present a more varied perspective. Only 40% of parents are satisfied or very satisfied, while 40% are dissatisfied or very dissatisfied, suggesting a notable disparity compared to school management’s evaluations. The parental assessments of school meal quality are more critical, with 55% rating it positively but 20% considering it poor or very poor. Regarding funding for school nutrition, 40% find it sufficient, while 60% express concerns, either rating it partially sufficient or insufficient. Cooperation with local governments is rated less favorably by parents, with only 50% giving it positive ratings compared to 25% rating it poorly. Nutritional standard adherence is viewed positively by most parents, with 80% indicating standards are often or always followed, though 20% believe they are infrequently or never followed. The frequency of sanitary inspections is considered regular by 60%, though 40% of parents perceive them as occasional or infrequent. Local government involvement in nutrition education is rated as low or very low by 40% of parents, reflecting higher expectations for such initiatives. Parents’ satisfaction with food supplier cooperation is mixed, with 40% rating it positively and 25% negatively, indicating room for improvement. Information on food allergies is rated positively by 55%, but 25% find it lacking. Healthy eating programs are generally well-implemented, with 70% of parents rating them as frequent, though 30% report infrequent or absent implementation. Local government response times to problems are seen as slow or very slow by 30% of parents, while only 35% rate the response as fast or very fast, indicating some dissatisfaction. Satisfaction with nutrition programs for children from low-income families is positive for 50% of parents, though 20% express dissatisfaction. Cafeteria staffing is perceived as partially sufficient or insufficient by 70% of parents, highlighting staffing concerns. Satisfaction with kitchen infrastructure is lower among parents, with 45% satisfied but 30% dissatisfied, indicating potential infrastructure issues. Finally, the adaptation of meals to dietary needs is rated as infrequent or insufficient by 55% of parents, suggesting a need for better dietary accommodation (Table 2).

### 3.3. Correlation of Management and Parent Satisfaction

The survey reveals significant differences in the evaluations of local government activities between school principals and parents. Principals generally provide more positive assessments. For instance, 25% of principals express high satisfaction with local government performance compared to only 15% of parents. Similarly, 70% of principals believe nutrition standards are always met, whereas only 50% of parents share this view. Regarding meal quality, 30% of principals rate it as very good, compared to just 20% of parents, highlighting a disparity in perceptions that may suggest a need for improved communication or meal quality. In terms of funding for school nutrition, 60% of principals find it sufficient, while only 40% of parents agree, with 30% of parents considering it inadequate versus 10% of principals. Cooperation with local governments is rated positively by 80% of principals, but only 50% of parents echo this sentiment, indicating a potential need for enhanced communication and collaboration between parents and local authorities. When it comes to meal adaptations for dietary needs, 75% of principals believe they are adequately adapted (40% always, 35% often), while only 45% of parents agree (20% always, 25% often), suggesting that parents are less satisfied with dietary accommodations, which may require further attention (Table 3).

High correlation (0.80 and higher):

Adherence to nutrition standards (0.961, *p* = 0.009), Frequency of sanitary inspections (0.963, *p* = 0.009), Allergy information (0.950, *p* = 0.013), Healthy eating programs (0.896, *p* = 0.040): high correlations indicate strong similarity in the evaluation of these aspects between school principals and parents, and low *p*-values indicate the statistical significance of these correlations.

Average correlation (0.50–0.79):

Nutrition programs for the poor (0.715, *p* = 0.174), Canteen staff resources (0.749, *p* = 0.145), Kitchen infrastructure (0.735, *p* = 0.157): the mean correlation suggests moderate agreement between principals and parents on these issues, although higher *p*-levels indicate that the correlations are not statistically significant.

Low correlation (less than 0.50):

Overall satisfaction (0.365, *p* = 0.546), Involvement in nutrition education (0.300, *p* = 0.624), Cooperation with suppliers (0.174, *p* = 0.780), Responsiveness to problems (0.360, *p* = 0.552), Adaptation of dietary meals (0.482, *p* = 0.411): the low correlation indicates significant differences in perceptions of these aspects between principals and parents. High *p*-levels further indicate that these differences are not statistically significant.

High correlations and low *p*-values in key areas such as adherence to nutrition standards, frequency of sanitary inspections, and allergy information indicate consistent positive evaluations of these aspects by principals and parents. However, lower correlations and higher *p*-levels in areas such as overall satisfaction, involvement in nutrition education, and responsiveness to problems suggest that these issues may need additional attention and improvement to better meet the expectations of both groups.

## 4. Discussion

The organization of school feeding by local governments is a controversial topic that requires careful analysis. In this discussion, we will compare the satisfaction of school management and parents in Poland and abroad based on the available research and scientific articles [9,10,11,12,13,14,15,16,17,18].

In Poland, the organization of school feeding by local governments is being systematically evaluated. Surveys conducted by the Foundation for Child Development indicate that most school principals express positive opinions about the role of local governments in providing school food. In a 2023 study, the Foundation noted that despite overall improvement, there are differences in the quality of services between different regions of the country [9].

A report by the Polish Dietetic Association, on the other hand, stresses that despite efforts, there are still problems related to the quality and variety of meals. In particular, the issue of tailoring meals to the individual dietary needs of students with allergies remains a challenge [10]. A review paper by Sienkiewicz et al. (2022) highlights that there is a need for increased funding and more flexible solutions that could improve the quality of nutrition [11].

In an international context, research findings show that school feeding systems vary widely from country to country. For example, a study conducted in Sweden by Johansson et al. (2021) shows that the school feeding system is well regarded by both principals and parents due to its high quality standards and effective management [12]. The Swedish system is characterized by high transparency and regular quality checks, which contributes to high levels of satisfaction [13].

In Finland, a study by Rantanen et al. (2022) confirms that the Finnish school feeding model is effective and highly regarded among parents and school principals. The model places a strong emphasis on healthy eating and the integration of local products, which has a positive impact on satisfaction [14].

In the United States, the situation is more diverse. According to a 2023 report by the National School Boards Association, there are significant differences in the quality of nutrition between states. Programs such as Farm to School in California are praised for their high quality and involvement of local communities, while other regions struggle with financial and logistical problems [15]. A study by Smith et al. (2022) highlights that a key challenge is to ensure equal access to healthy meals in all schools, which is difficult to achieve due to regional differences [16].

Comparing Polish and foreign perspectives, it can be seen that in countries such as Sweden and Finland, school feeding systems are more integrated and better funded, resulting in higher levels of satisfaction among principals and parents. In Poland and the US, funding challenges and regional inequalities have a significant impact on nutrition quality. In Poland, in particular, further reforms and increased funding are needed to meet the expectations of parents and school principals [17,18].

To reduce the gap in perceptions of nutrition quality between school management and parents, it is recommended that transparency be increased in communication regarding nutrition standards and procedures. Schools should regularly inform parents about the quality of meals, procedures for complying with nutrition standards, and the results of sanitary inspections. They can also hold informational meetings and open days at school cafeterias so that parents can see for themselves the conditions and process of preparing meals. Given parents’ dissatisfaction with schools’ cooperation with suppliers, it is recommended that food suppliers be regularly monitored and evaluated for product quality and compliance with parents’ expectations. Schools should consider putting in place feedback mechanisms that allow parents to comment on suppliers. In addition, it is important for schools to step up efforts to better tailor meals to children’s dietary needs, especially in the context of food allergies and intolerances. In response to parents’ criticism of schools’ involvement in nutrition education, it is recommended that educational activities in this area be increased. Nutrition education programs should be more comprehensive and involve not only students but also parents. Consideration could be given to introducing regular workshops and meetings with nutritionists aimed at promoting healthy eating habits in students’ homes. This will make parents better informed and more involved in their children’s educational process, which could help improve their satisfaction with the quality of nutrition in schools.

### Strengths and Limitations

The conducted survey exhibits several key strengths that enhance its validity and reliability. One significant advantage is the sample size, which includes 200 school principals and 1000 parents of students, ensuring the representativeness of the findings and providing broad coverage of the examined issue. By incorporating the perspectives of both groups, the survey offers a comprehensive assessment of the implementation of nutrition-related tasks in educational institutions. Another strength lies in the wide range of aspects evaluated, from meal quality to supplier cooperation and nutrition education, which contributes to a detailed understanding of the school nutrition system. Furthermore, the application of statistical analyses, such as Pearson correlation and tests of statistical significance, allowed for the precise identification of differences in the evaluations provided by principals and parents, as well as the determination of their statistical significance, thus strengthening the robustness of the results.

However, certain limitations must be considered when interpreting the findings. Firstly, the nationwide scope of the survey does not account for regional variations that may influence local governments’ implementation of nutrition policies, potentially leading to oversimplifications in representing the actual situation across different regions. Additionally, the subjective nature of the assessments from both school management and parents introduces variability in the results due to individual experiences. The risk of non-response bias also exists, as some respondents may have opted not to participate, potentially impacting the comprehensiveness of the satisfaction assessment with school food services. Furthermore, while the survey identifies correlations between the evaluations of principals and parents, it does not clarify the underlying reasons for these differences, nor does it directly consider external factors such as local government policies or schools’ financial conditions. Lastly, the possibility of socially desirable responses cannot be ruled out, which may result in the underreporting of critical opinions or the overreporting of positive assessments, thereby imposing some constraints on the reliability of the findings.

Although the survey focused on the evaluations of school principals and parents, it also seems important to include the perspectives of other stakeholders, such as teachers, students, and local government representatives. Teachers’ perspectives could shed light on the day-to-day organization of meals and students’ involvement in nutrition education, while students’ opinions could provide valuable information on the taste, appeal, and availability of meals. Including local government officials would provide a better understanding of the challenges of funding and organizing nutrition programs.

## 5. Conclusions

1. The survey results show moderate agreement between school management and parents in assessing local governments’ implementation of nutrition-related tasks. While there is general alignment on issues like adherence to nutrition standards and sanitary inspections, parents report lower overall satisfaction, likely due to higher expectations or differing perceptions of service quality.
2. School principals generally view the quality and organization of nutrition more positively than parents. The high correlation in ratings for meal quality and adherence to standards suggests principals have a more favorable view, while parents are more critical. This highlights the need for better communication with parents about school procedures to reduce these discrepancies.
3. Parents express greater dissatisfaction with operational aspects, such as cooperation with suppliers, allergy information, and nutrition education, indicating a desire for more transparency and involvement. These statistically significant differences suggest that better communication and enhanced nutrition education are needed to align expectations between parents and schools.

## Figures and Tables

**Table 1:** Satisfaction level of school management by satisfaction scale.

Item	Very Satisfied	Satisfied	Neutral	Disgruntled	Very Dissatisfied
Overall satisfaction	25%	40%	20%	10%	5%
Quality of meals	30%	50%	15%	4%	1%
Availability of financing	60%	30%	-	10%	-
Cooperation with local governments	35%	45%	15%	4%	1%
Adherence to nutritional standards	70%	25%	5%	-	-
Frequency of sanitary inspections	80%	15%	5%	-	-
Commitment to nutrition education	20%	40%	30%	10%	-
Cooperation with suppliers	40%	45%	10%	4%	1%
Allergy information	50%	35%	10%	4%	1%
Healthy eating programs	65%	25%	8%	2%	-
Response to problems	25%	45%	20%	8%	2%
Nutrition programs for the poor	30%	50%	15%	4%	1%
Human resources in canteens	55%	30%	-	15%	-
Kitchen infrastructure	30%	40%	20%	8%	2%
Adaptation of dietary meals	40%	35%	20%	5%	-

**Table 2:** Parents’ satisfaction level by satisfaction scale.

Item	Very Satisfied	Satisfied	Neutral	Disgruntled	Very Dissatisfied
Overall satisfaction	15%	25%	20%	25%	15%
Quality of meals	20%	35%	25%	15%	5%
Availability of financing	40%	30%	-	30%	-
Cooperation with local governments	20%	30%	25%	15%	10%
Adherence to nutritional standards	50%	30%	15%	5%	-
Frequency of sanitary inspections	60%	25%	15%	-	-
Commitment to nutrition education	10%	20%	30%	25%	15%
Cooperation with suppliers	15%	25%	35%	15%	10%
Allergy information	30%	25%	20%	15%	10%
Healthy eating programs	40%	30%	20%	10%	-
Response to problems	10%	25%	35%	20%	10%
Nutrition programs for the poor	20%	30%	30%	15%	5%
Human resources in canteens	30%	40%	-	30%	-
Kitchen infrastructure	20%	25%	25%	20%	10%
Adaptation of dietary meals	20%	25%	30%	25%	-

**Table 3:** Correlation of management and parent satisfaction by satisfaction scale items.

Item	Correlation Level	*p*-Value
Overall satisfaction	0.365	0.546
Quality of meals	0.872	0.054
Availability of financing	0.839	0.076
Cooperation with local governments	0.810	0.096
Adherence to nutritional standards	0.961	0.009
Frequency of sanitary inspections	0.963	0.009
Commitment to nutrition education	0.300	0.624
Cooperation with suppliers	0.174	0.780
Allergy information	0.950	0.013
Healthy eating programs	0.896	0.040
Response to problems	0.360	0.552
Nutrition programs for the poor	0.715	0.174
Human resources in canteens	0.749	0.145
Kitchen infrastructure	0.735	0.157
Adaptation of dietary meals	0.482	0.411

## Data Availability

The data presented in this study are available upon request from the corresponding author. The data are not publicly available due to restrictions that apply to the availability of these data.
